# Insights into Diphthamide, Key Diphtheria Toxin Effector

**DOI:** 10.3390/toxins5050958

**Published:** 2013-05-03

**Authors:** Wael Abdel-Fattah, Viktor Scheidt, Shanow Uthman, Michael J. R. Stark, Raffael Schaffrath

**Affiliations:** 1 Institut für Biologie, FG Mikrobiologie, Universität Kassel, Kassel D-34132, Germany; E-Mails: wael@uni-kassel.de (W.A.-F.); scheidtviktor@yahoo.de (V.S.); 2 Department of Genetics, University of Leicester, Leicester LE1 7RH, UK; E-Mail: uu4@le.ac.uk; 3 Centre for Gene Regulation & Expression, University of Dundee, Dundee, DD1 5EH, Scotland; E-Mail: m.j.r.stark@dundee.ac.uk

**Keywords:** diphtheria toxin, sordarin, diphthamide biosynthesis, *DPH1-DPH7* genes

## Abstract

Diphtheria toxin (DT) inhibits eukaryotic translation elongation factor 2 (eEF2) by ADP-ribosylation in a fashion that requires diphthamide, a modified histidine residue on eEF2. In budding yeast, diphthamide formation involves seven genes, *DPH1-DPH7*. In an effort to further study diphthamide synthesis and interrelation among the Dph proteins, we found, by expression in *E. coli* and co-immune precipitation in yeast, that Dph1 and Dph2 interact and that they form a complex with Dph3. Protein-protein interaction mapping shows that Dph1-Dph3 complex formation can be dissected by progressive *DPH1* gene truncations. This identifies N- and C-terminal domains on Dph1 that are crucial for diphthamide synthesis, DT action and cytotoxicity of sordarin, another microbial eEF2 inhibitor. Intriguingly, *dph1* truncation mutants are sensitive to overexpression of *DPH5*, the gene necessary to synthesize diphthine from the first diphthamide pathway intermediate produced by Dph1-Dph3. This is in stark contrast to *dph6* mutants, which also lack the ability to form diphthamide but are resistant to growth inhibition by excess Dph5 levels. As judged from site-specific mutagenesis, the amidation reaction itself relies on a conserved ATP binding domain in Dph6 that, when altered, blocks diphthamide formation and confers resistance to eEF2 inhibition by sordarin.

## 1. Introduction

**Figure 1:**
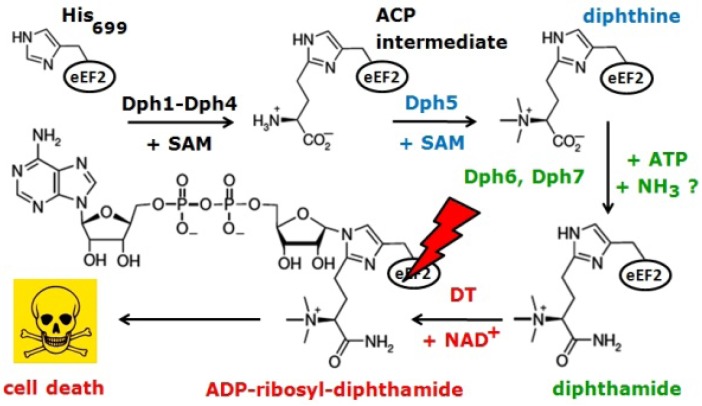
Diphthamide synthesis on yeast translation elongation factor 2 (eEF2) and ADP-ribosylation by diphtheria toxin (DT). For details, see text.

Diphthamide (2-[3-carboxyamido-3-(trimethylamino)-propyl]-histidine) is an unusual modified histidine residue in eukaryotic translation elongation factor 2 (eEF2). It is the target of diphtheria toxin (DT) from *Corynebacterium diphtheriae* [1], and ADP-ribosylation of diphthamide by DT blocks protein synthesis by inhibiting eEF2 function. eEF2 in the budding yeast *Saccharomyces cerevisiae* contains diphthamide at position 699 (Figure 1) and like eEF2 from other eukaryotes can be ADP-ribosylated and inhibited by DT.

Studies in yeast have proved very useful for investigating diphthamide synthesis, which operates through a multi-step pathway that involves seven genes (*DPH1-DPH7*) [2,3,4,5,6]. It starts with the transfer of the 3-amino-3-carboxypropyl (ACP) group from S-adenosylmethionine (SAM) to the imidazole ring of His_699_ on eEF2 (Figure 1). This step depends on diphthamide synthesis factors Dph1-Dph4 and generates the ACP-modified intermediate of His_699_ (Figure 1) in a fashion that involves radical SAM and Fe-S enzyme chemistry [2,7,8,9]. Next, the latter undergoes trimethylation yielding diphthine in a reaction catalysed by diphthine synthase Dph5 and requiring SAM as methyl donor (Figure 1) [10,11]. Finally, diphthine amidation generates the end product diphthamide in an energy-dependent process involving Dph6 and Dph7 (Figure 1) [4,5,6]. The diphthamide imidazole ring is the site for NAD^+^-dependent ADP-ribosylation by DT, a modification (ADP-ribosyl-diphthamide), that irreversibly inactivates the translation function of eEF2 and leads to cell death (Figure 1) [12,13]. However, the intermediate diphthine is also a very weak substrate for inhibitory ADP-ribosylation [14]. Intriguingly, another eEF2 inhibitor, sordarin, which unlike DT blocks the eEF2-ribosome complex in yeast and fungi, is also dependent on diphthamide formation on eEF2. This is why yeast *dph1-dph7* mutants are resistant to growth inhibition by sordarin [15,16].

In a further effort to study diphthamide formation in yeast and analyze interrelationships among individual components of the pathway required for diphthamide synthesis, we examine here genetic as well as biochemical interactions between the products of the *DPH1*, *DPH2*, *DPH3*, *DPH5* and *DPH6* genes. 

## 2. Results and Discussion

### 2.1. Dph1 Protein-Protein Interactions

**Figure 2:**
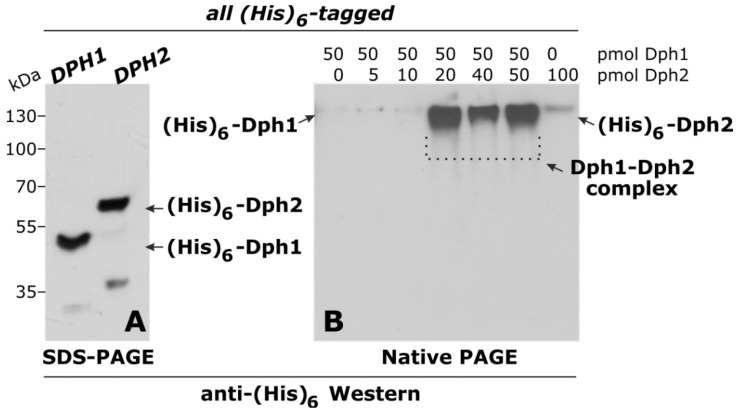
(His)_6_-tagged Dph1 and Dph2 expressed from *E. coli* interact with each other *in vitro*. (**A**) Anti-(His)_6_ Western blot following denaturing conditions (12% SDS-PAGE). (**B**) Western blot under native conditions (10% native PAGE, 0.5 × TBE).

In an effort to further study diphthamide formation in yeast, we examined protein-protein interactions between Dph1, Dph2 and Dph3. Upon expressing (His)_6_-tagged versions of either Dph1 or Dph2 in *E. coli*, both proteins were detectable with anti-(His)_6_ antibodies under denaturing conditions on Western blots (Figure 2A). Under native conditions, however, Dph1 and Dph2 alone were hardly detectable, while mixtures of both proteins gave rise to strong signals in anti-(His)_6_ Western blots (Figure 2B). This implies that when in solution alone and in native conformation the (His)_6_ tag on each protein is buried and undetectable, whereas the (His)_6_ tags become detectable under native conditions when Dph1 and Dph2 interact with one another and form a protein complex. Such a Dph1-Dph2 heterodimer is well in line with *in vivo* interactions seen between Dph1 and Dph2 in yeast and mammalian cell systems [2,15] and suggests that Dph1-Dph2 complex formation might be physiologically relevant for diphthamide synthesis. 

**Figure 3:**
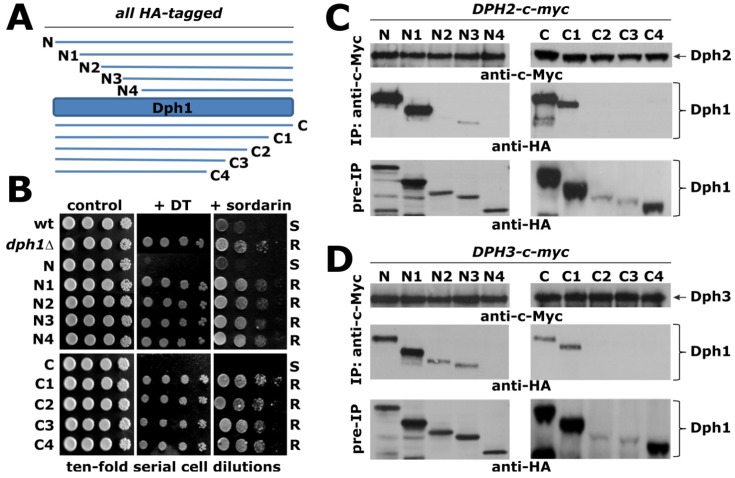
Use of DT and sordarin as diagnostic tools to map Dph1 regions crucial for Dph2 and Dph3 interaction. (**A**) Diagram illustrating the N- and C-terminal Dph1 truncation sets (Figure S1) used to study Dph1 function and interaction profiles. (**B**) DT and sordarin sensitivity assays. Serial cell dilutions of wild-type (wt), *DPH1* deletant (*dph1Δ*) and the strains indicated in panel A were grown in the absence (control) or presence of DT or sordarin. ‘S’ and ‘R’ denote sensitive and resistant traits, respectively. (**C**, **D**) Anti-c-Myc co-immune precipitation (IP) assays to study Dph1-Dph2 and Dph1-Dph3 protein-protein interactions. The presence of c-Myc-tagged Dph2 (panel C), Dph3 (panel D), the HA-tagged full-length Dph1 (N, C) and the N- and C-terminal truncation variants of Dph1 in the IPs were monitored by anti-c-Myc and anti-HA Western blots. In addition, the content of full-length and truncated forms of HA-tagged Dph1 was checked by immune blots in the inputs (pre-IP). The positions of Dph2, Dph3 as well as full-length and truncation forms of Dph1 are indicated by arrows.

Previously, it was shown that in yeast, both Dph1 and Dph2 copurify with Dph3 potentially as part of a multimeric complex [15,17]. To gain further insights into formation of such a Dph1-Dph2-Dph3 complex, we focused on Dph1 and sought to identify regions crucial for interactions with Dph2 and/or Dph3 *in vivo*. Using PCR protocols for genomic manipulations [18], HA epitope-tagged versions of full-length Dph1 and progressive N- or C-terminal truncations (Figure S1) were generated in strains expressing c-Myc-tagged forms of Dph2 or Dph3 (Figure 3A, C and D). Here, the rationale was to identify non-functional Dph1 truncation variants on the basis of resistance to DT and to sordarin (Figure 3B), both traits associated with Dph1 defects [15,17], and then to examine their Dph2 and Dph3 interaction profiles using anti-c-Myc co-immune precipitation assays (Figure 3C and D).

As illustrated by the N-terminal truncation set (Figure 3C and D), which removed 60 (N1), 90 (N2), 120 (N3) and 150 (N4) amino acid residues from HA-tagged Dph1, the truncations N2-N4 and full-length Dph1 were detected at similar levels together with some low-abundance degradation products in anti-HA Western blots. N1 levels, however, were slightly increased. N1 through N4 conferred DT resistance and protection against growth inhibition by sordarin, both phenotypes the truncation mutants share with a *DPH1* deletion mutant (Figure 3B).

This indicates that the N1-N4 truncations of Dph1 are non-functional and have a defect in diphthamide synthesis. Intriguingly, this defect does not appear to result from their inability to form a Dph1-Dph2-Dph3 complex since the HA-tagged N1 truncation of Dph1 (and to a lesser extent both the N2 and N3 truncations) could be co- precipitated with c-Myc-tagged Dph3 (Figure 3D). Similary, the HA-tagged N1 truncation of Dph1 was found to interact with c-Myc-tagged Dph2 (Figure 3C). These protein-protein interaction profiles rather suggest that a diphthamide defect can occur in spite of the intact Dph1-Dph2-Dph3 interactions. Presumably, the complex which is formed in the presence of the N1 truncation is no longer enzymatically active and therefore, unable to support formation of the ACP intermediate during diphthamide synthesis (Figure 1).

Analysis of the C-terminal Dph1 truncation set revealed that removal of the last 30 residues (C1) neither impacted on Dph1 stability nor on interaction with Dph2 or Dph3, while larger truncations of 60 (C2) and 90 (C3) residues caused severe Dph1 instability and as a result, induced loss of Dph2 and Dph3 (Figure 3C and D) interactions. Although truncation C1 supported Dph2 and Dph3 interactions (Figure 3C and D), it nonetheless conferred resistance to growth inhibition by DT and sordarin (Figure 3B). This reinforces the findings from the above N-terminal truncation set and shows that albeit crucial for functioning in the first diphthamide pathway step, assembly of the Dph1-Dph2-Dph3 complex *per se* is not sufficient to initiate diphthamide synthesis (Figure 1). Our *DPH1* truncation analysis is consistent with a mutagenesis report on the *DPH2* gene from Chinese hamster ovary cells [19]. Here, it was shown that deletion of 91 amino acid residues from the C-terminus of DPH2 was sufficient to block diphthamide synthesis and cause DT resistance, yet the truncated DPH2 was able to co-purify and interact with rodent DPH1 [19]. 

Presumably, though dispensable for Dph2 and Dph3 interaction, the extreme N- and C-termini of Dph1 are required to maintain the complex enzymatically competent and hence to support formation of the ACP intermediate (Figure 1). In the light of recent reports from archaea showing that ACP generation from SAM requires conserved cysteine residues (Figure S1) for the assembly of a [4Fe-4S] cluster in Dph2 [7,8], the N- or C-termini of Dph1 may be required for proper Fe-S and radical SAM enzyme chemistry of the Dph1-Dph2-Dph3 complex.

### 2.2. DPH5 Overexpression Toxicity Effects

**Figure 4:**
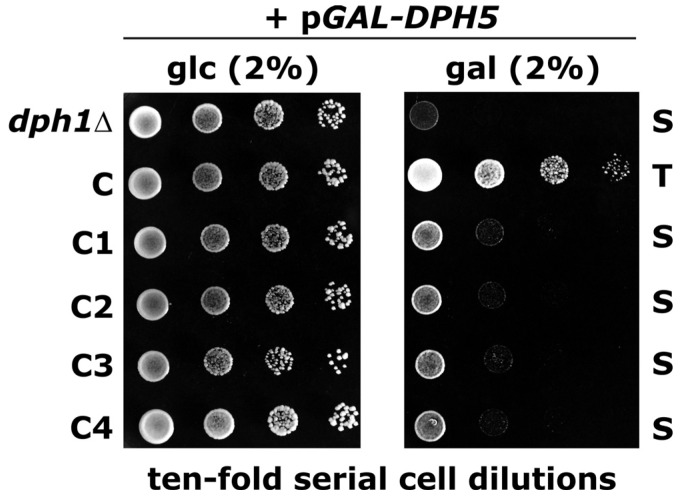
Overexpression of *DPH5* is growth inhibitory to *dph1* truncation and deletion mutants. Strains with the indicated genetic backgrounds (see Figure 3) and maintaining plasmid p*GAL-DPH5* for galactose inducible overexpression of Dph5 were serially diluted and spotted onto glucose (2% glc) and galactose (2% gal) media to assay their response to *DPH5* overexpression. Unaltered tolerance (T) and sensitive (S) responses are indicated.

Recently, we showed that *DPH5* overexpression from a galactose-inducible promoter is highly detrimental to the growth of *dph* mutants with a block at the first step of the diphthamide synthesis (Figure 1), but had little or no effect on wild-type, *dph5* or *dph6* cells [6]. To ask whether or not *DPH5* overexpression toxicity in the absence of ACP intermediate formation requires assembly of the Dph1-Dph2-Dph3 complex, we assayed the growth performance of the C1-C4 truncation mutants (Figure 3A) on galactose following transformation with the inducible *DPH5* expression vector. As illustrated in Figure 4, growth of all four C-terminal *DPH1* deletion constructs including variant C1, which allows for Dph1-Dph2-Dph3 interactions (Figure 3C and D), was as sensitive to galactose as a *dph1Δ* null-mutant which was included as an internal control. Thus, the Dph1-Dph3 complex formed in the C1 truncation mutant is not able to protect against growth inhibition by higher-than-normal levels of Dph5, suggesting that it is lack of ACP intermediate formation which determines *DPH5* overexpression toxicity. Intriguingly, we found previously that *DPH5* cytotoxicity goes hand in hand with enhanced Dph5-eEF2 interaction profiles observed in *dph1* cells but not in wild-type or *dph5* cells [6]. Presumably, enhanced binding of Dph5 to inappropriately modified or unmodifed eEF2 may be detrimental to the function of the translation factor and, as a consequence, is inhibitory to the growth of the *dph1* truncation and deletion mutants. 

### 2.3. DPH6 Mutagenesis

**Figure 5:**
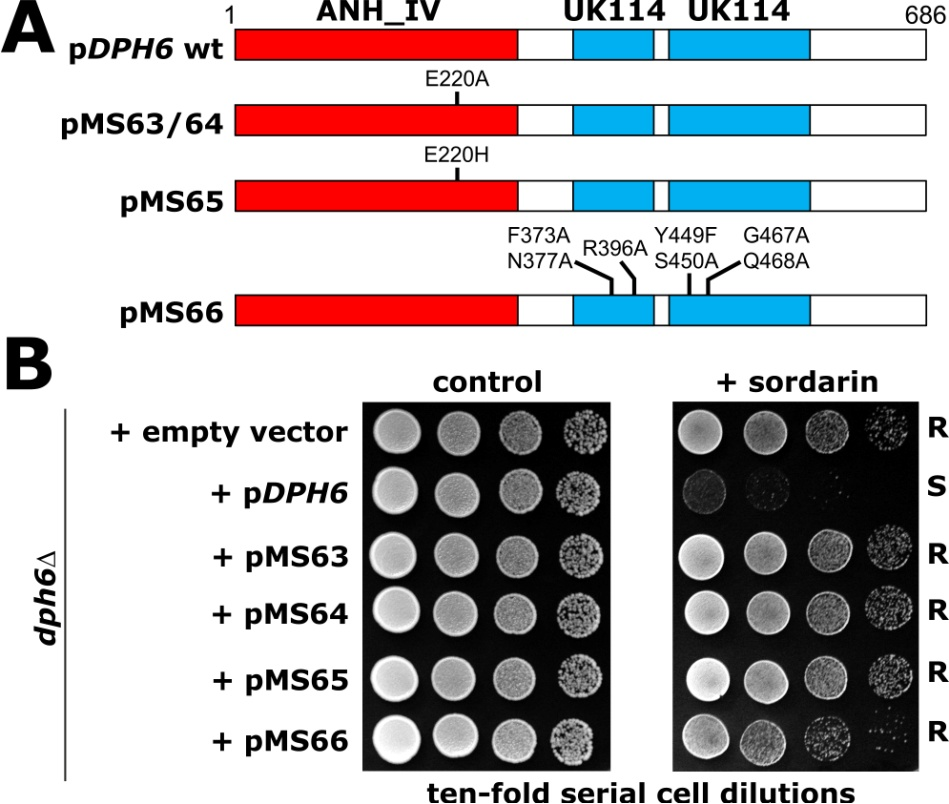
*DPH6* mutagenesis identifies domains in Dph6 that are essential for its function in sordarin sensitivity and dipthamide synthesis. (**A**) Diagram showing the *DPH6* wild-type and mutant constructs tested in (**B**), indicating the Alpha_ANH_like_IV (ANH_IV: red) and YjgF-YER057c-UK114 (UK114: blue) domains and the position of point mutations. (**B**) Ten-fold serial cell dilutions of a *dph6Δ* deletion strain carrying the constructs shown in (**A**) or the corresponding empty vector pSU6 were grown onto plates with or without sordarin. ‘S’ and ‘R’ denote sensitive and resistant traits, respectively.

Consistent with its functioning as a diphthine amidase in the last step of diphthamide synthesis (Figure 1), the *DPH6* gene product contains three enzymatically relevant and conserved protein domains (Figure 5A). The amino-terminal ANH_IV (Alpha_ANH_like_IV) domain is predicted to bind ATP via a highly conserved sequence motif [E_215_GG(D/E)XE_220_] [6]. We generated two *dph6* alleles encoding single amino acid substitutions in this region (E220A; E220H) and tested their functionality by monitoring ability to complement the sordarin resistance phenotype of a yeast *dph6Δ* null-mutant. As illustrated in Figure 5B, both alterations inactivate the function of Dph6, demonstrating that the ANH_IV domain is critical for completion of diphthamide synthesis and sordarin sensitivity. The C-terminus of Dph6 contains two YjgF-YER057c-UK114 protein family domains (UK114: Figure 5A) that may have enamine/imine deaminase activity and be used to generate ammonia for diphthine amidation and diphthamide formation [6]. Truncation of Dph6 before these domains leads to an inactive protein [6], but as a more stringent test of the requirement for these domains we made point mutations in key, conserved residues in the context of the full-length protein. This confirmed that the UK114 domains in Dph6 are important for sordarin sensitivity and that, on its own, the ANH_IV domain is non-functional (Figure 5B). Taken together, this suggests a direct, ATP-dependent role for Dph6 in the final amidation step of diphthamide synthesis (Figure 1), a notion that has recently been confirmed by an *in vitro* reconstitution assay showing that Dph6 has ATP-dependent diphthine amidase activity [5]. It will be interesting to determine the precise role of the UK114 domains in Dph6 and to test whether they might provide ammonium as the direct amide source for the final *in vivo* diphthamide synthesis step.

## 3. Experimental Section

### 3.1. DPH1 and DPH2 Overexpression in *E. coli*

For bacterial (His)_6_-Dph1 and (His)_6_-Dph2 overproduction, we chose the pETDuet-1 (Novagen) expression vector and placed the yeast genes *DPH1* and *DPH2* under control of the T7 promoter/*lac* operator region using *Hin*dIII/*Not*I (*DPH1*) and *Sal*I/*Not*I (*DPH2*) directional cloning. Both inserts were derived from PCR using BG1805 plasmid templates previously described [20] and primers specific to *DPH1* (*Hin*dIII fw: 5′-CATAAGCTTATGAGTGGCTCTACAGAATCTAAA-3′ and *DPH1 Not*I rv: 5′-CATGCGGCCGCTTCAATCGCATGTTTCGGAGTTTCC-3′) or *DPH2* (*DPH2 Sal*I fw: 5′-CATGTCGACATGGAAGTTGCACCGGCCTTA-3′ and *DPH2 Not*I rv: 5′-CATGCGGCCGCTTTGTTTTCCTTTTTCATAG-3′). Protein production from the resulting expression vectors, pSV8 (*DPH1*) and pSV9 (*DPH2*), was done in *E. coli* strain BL 21DE3 Rosetta with 1 mM IPTG induction. After cell lysis, (His)_6_-Dph1 and (His)_6_-Dph2 protein purification involved wash and elution steps in the presence of 20 mM and 500 mM imidazole followed by a second elution with 100 mM EDTA and dialysis against 20% (w/v) glycerol. Following protein separation under denaturing (12% SDS-PAGE) or native (10% PAGE in 0.5 × TBE) conditions, standard Western blots were run using anti-(His)_6_ antibodies (Santa Cruz Biotechnology).

### 3.2. DPH1 Truncation Mutants

N-terminal and C-terminal HA-tagging of *DPH1* full-length (N and C) and truncation alleles (N1–N4 and C–C4: Figure S1) was performed according to previously published *in vivo* PCR-based epitope tagging protocols using appropriate S3/S2 [18] or F4/R3 [21] primer pair combinations (Table 1). Tagged gene products were confirmed by Western blot detection with anti-HA antibodies (Santa Cruz Biotechnology); parallel *DPH2-c-myc* and *DPH3-c-myc* taggings and co-immune precipitation studies were as previously reported [15]. Diphtheria toxin (DT) growth assays *in vivo* involved expression of the toxin’s cytotoxic ADP ribosylase fragment from vector pSU8 essentially as previously described [6]. For sordarin assays, the truncation mutants were cultivated at 30 °C on yeast peptone complete medium supplemented with 10 µg/mL sordarin sodium salt from *Sordaria araneosa* (Sigma-Aldrich). *DPH5* overexpression toxicity assays used the galactose inducible plasmid p*GAL-DPH5* and were essentially as described [6]. 

**Table 1:** Primers used for *DPH1* truncations and HA-tagging.

Name	Sequence (5’➔3’)	Use
S2-*DPH1*	GAATATGATACTAACTATTTATACATATGTAACAGGAAGACAAGTGACAACAAAAACTATTTAAAATCGATGAATTCGAGCTCG	*DPH1* C-terminal HA tagging
S3-*DPH1*	ATCCAATGGATTATTACGAAGCTAAAGGATACGGGCGTGGGGAAACTCCGAAACATGCGATTGAACGTACGCTGCAGGTCGAC	*DPH1* C-terminal HA tagging
S3.1-*DPH1*	TCAATAAACCACTATTAACACCATATGAGGCTAGTGTCTTACTAAAGAAACGTACGCTGCAGGTCGAC	*DPH1* HA tagging & C1-truncation
S3.2-*DPH1*	TTATTCTAAGTGAAGTTTTTCCCCAAAAGCTCGCAATGTTCGATCAAATTGATGTTTTTGTTCAGCGTACGCTGCAGGTCGAC	*DPH1* HA tagging & C2-truncation
S3.3-*DPH1*	GTAGACAAGGTAATTTAAACACTGTAAAAAACTTGGAAAAAAACCTGATCCGTACGCTGCAGGTCGAC	*DPH1* HA tagging & C3-truncation
S3.4-*DPH1*	TCACTAGAGAAGGATACGATCAAAAGCAACTCGTGGAAGTTAGAGCAGAGGCCATTGAAGTCGCTCGTACGCTGCAGGTCGAC	*DPH1* HA tagging & C4-truncation
F4- *DPH1*	AGAAATATAAATTCCTCATCCTGTGTTATAGAGAATCTTGGTGTTATCATTATAGTTCAGAAGTGGAATTCGAGCTCGTTTAAAC	*DPH1* N-terminal HA tagging
R3- *DPH1*	CCAATAAATCTTCTTCTTGGTTGTTTTTTAGATTCTGTAGAGCCACTCATGCACTGAGCAGCGTAATCTG	*DPH1* N-terminal HA tagging
R3.1- *DPH1*	TTGTAGTTAGAGGGCAATAATTTGATGGCTTCATTCAACTCTTTGTCATTGCACTGAGCAGCGTAATCTG	*DPH1* HA tagging & N1-truncation
R3.2- *DPH1*	TCACTTATAATCAATGAGTAAATCAGCAAACCTTCAGGCATCTGTAGGGCTATTCTTTTAGCATTGCACTGAGCAGCGTAATCTG	*DPH1* HA tagging & N2-truncation
R3.3- *DPH1*	TCATCAATACAGCATGCACCATAAGACACATCCCCCATTACTAGAGTTTCGCACTGAGCAGCGTAATCTG	*DPH1* HA tagging & N3-truncation
R3.4- *DPH1*	AGTACTTTAATCTTTGTAACGTCAATAGGAACTAAACACGAATGAGCGTAGCACTGAGCAGCGTAATCTG	*DPH1* HA tagging & N4-truncation

### 3.3. DPH6 Mutagenesis

p*DPH6* wt (pSU6) was generated by insertion of *DPH6* into YCplac111 as previously described [6]. To generate the E220A and E220H *dph6* mutants, pSU6 was digested with *Age*I and *Bsm*BI and the small *DPH6* fragment replaced by an identical synthetic fragment (Integrated DNA Technologies) carrying the corresponding mutations, generating pMS63/64 (E220A) and pMS65 (E220H) (Figure 5A). The *Age*I-*Bsm*BI region contributed by the synthetic DNA was verified by DNA sequencing. pMS66 was similarly generated and verified, but by replacing the *Bsm*BI-*Spe*I fragment within *DPH6* with an equivalent synthetic fragment carrying the mutations F373A, N377A, R396A, Y449F, S450A, G467A, Q468A in residues conserved in YjgF-YER057c-UK114 family proteins (Figure 5A).

## 4. Conclusions

We conclude that Dph1 and Dph2 interact *in vitro* and that the first step of diphthamide formation on eEF2 requires a complex formed *in vivo* between Dph1, Dph2 and Dph3. Blocked diphthamide synthesis on eEF2 associates with cell growth inhibition by excess levels of Dph5, the synthase required to form the second diphthamide pathway intermediate diphthine. Diphthine amidation by diphthamide synthetase requires ANH_IV and UK114 protein domains on Dph6 that are enzymatically significant and confer sensitivity to sordarin, a diphthamide-dependent eEF2 inhibitor with antifungal properties.

## Supplementary Files

Supplementary File 1 Supplementary Information (PDF, 683 KB)
