# Supplementary material for: Insights into Diphthamide, Key Diphtheria Toxin Effector
Source: Toxins (Basel). 2013 May 3;5(5):958–68. doi: 10.3390/toxins5050958 (PMC3709272; doi:10.3390/toxins5050958)

Supplementary Information

Figure S1. Sequence alignment of Dph1 and orthologs. Archaeal PhDph2 (*P. horikoshii*), mammalian chinese hamster (*C. griseus*) CgDph2, human HsDph1 and HsDph2, and *S. cerevisiae* ScDph2 are included for comparison of conserved regions. The highest degree of conservation between the six sequences is marked in dark blue. The three conserved cysteine residues in PhDph2, which bind the iron-sulfur cluster (Cys59, Cys163 and Cys287) are marked with a red triangle [7,8]. The position of N-terminal truncations (N1-N4) as well as C-terminal truncations (C1-C4) of ScDph1 are highlighted. Sequence alignment was performed using Jalview.

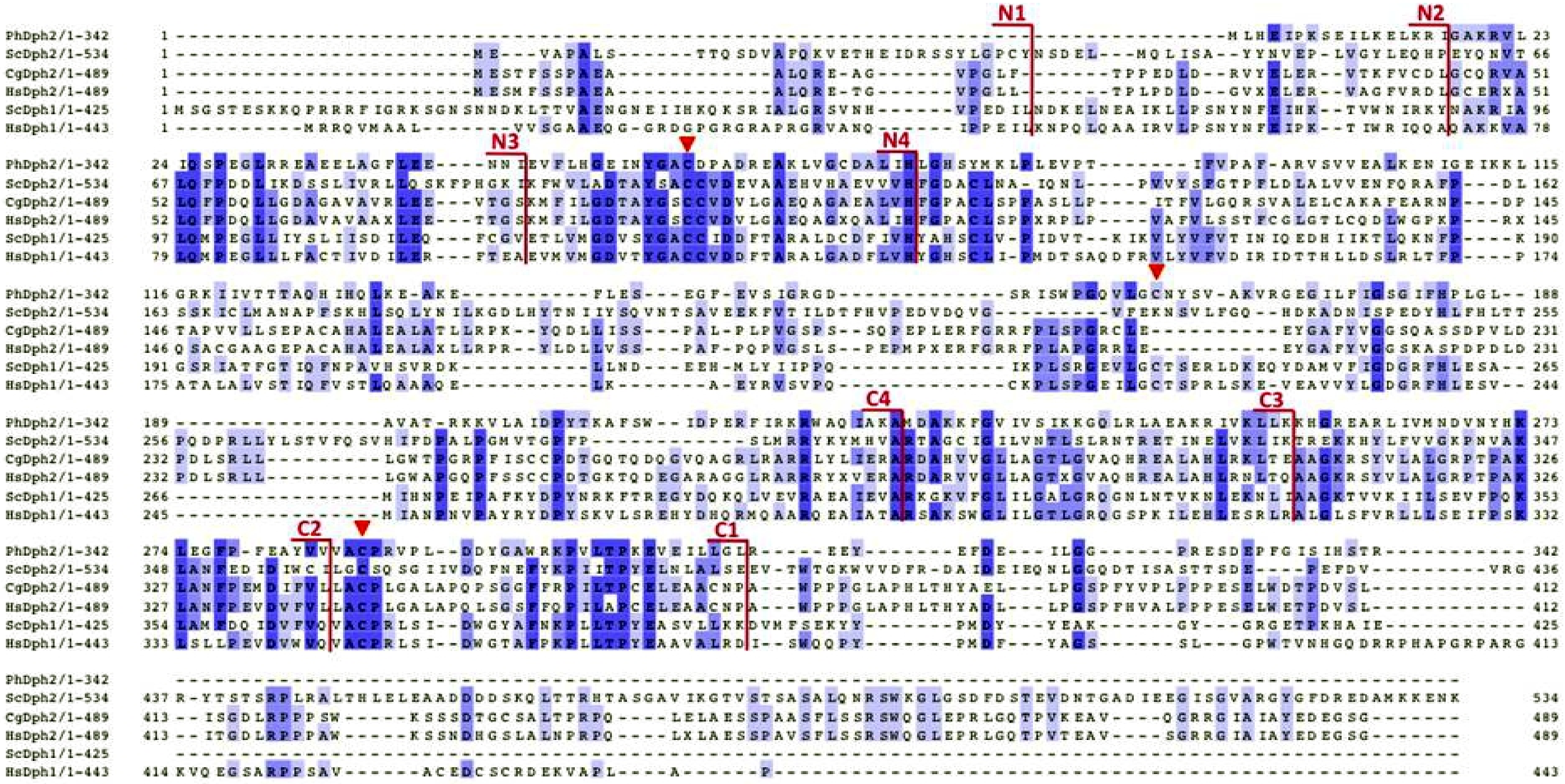

Supplement: Supplementary File 1 — Supplementary Information (PDF, 683 KB) [file toxins-05-00958-s001.pdf]
